# Supplementary material for: ZNF827 is a single-stranded DNA binding protein that regulates the ATR-CHK1 DNA damage response pathway
Source: Nat Commun. 2024 Mar 12;15:2210. doi: 10.1038/s41467-024-46578-0 (PMC10933417; doi:10.1038/s41467-024-46578-0)
Supplement: Supplementary file 1 — Supplementary Information [file 41467_2024_46578_MOESM1_ESM.pdf]

Supplementary Figure 1.

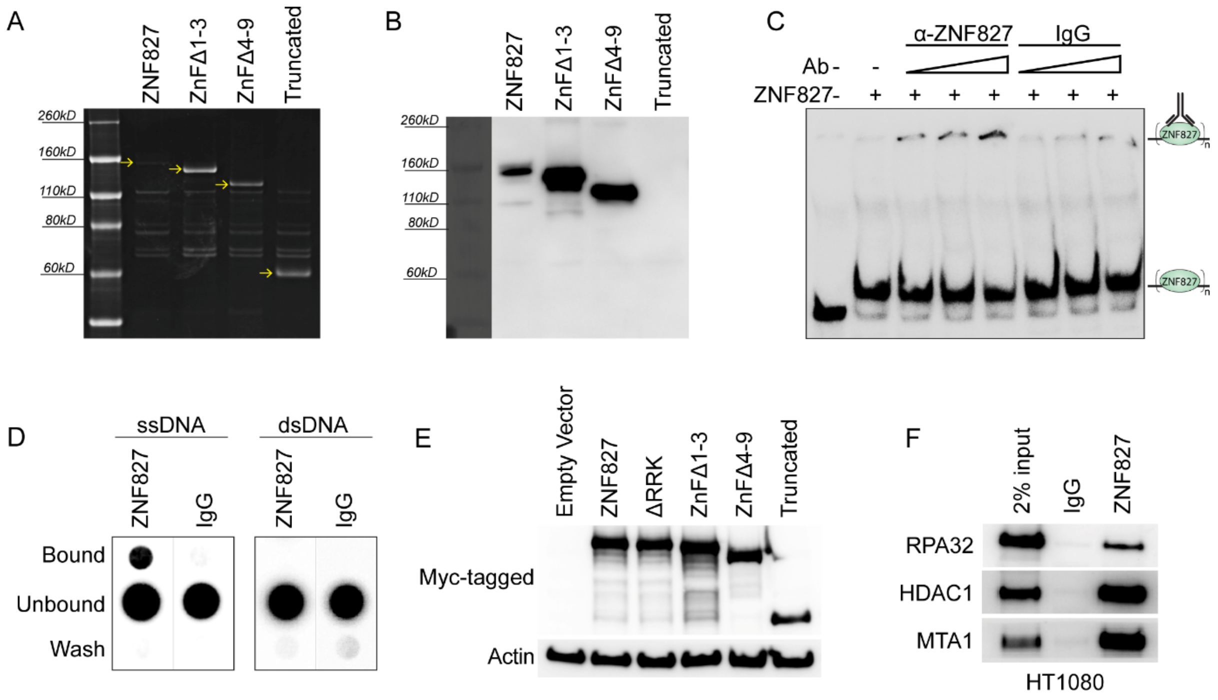

Supplementary Figure 1. ZNF827 protein expression and purification. Related to Figure 1. Protein purification in HEK 293T cells transfected for 48 h with HaloTag® ZNF827 and ZNF827 ZnF mutant constructs. **(A)** Coomassie gel of purified untagged ZNF827 and ZNF827 ZnF mutants after protein purification by HaloTag®. **(B)** Western blot analysis of purified untagged ZNF827 and ZNF827 ZnF mutants after protein purification by HaloTag®. The truncated mutant could not be detected by the ZNF827 antibody due to loss of epitope. **(C)** Super-shift electrophoretic mobility shift assay (EMSA) using γ-^{32}P radiolabelled single-stranded pentaprobes incubated with purified ZNF827 protein and up to 1.8 μg of either IgG control or ZNF827 antibody. **(D)** Detection of immunoprecipitated DNA using γ-^{32}P radiolabelled probe following incubation of single-stranded or double-stranded pentaprobes with purified ZNF827 and immunoprecipitation with direct ZNF827 antibody or IgG control. **(E)** Western blot analysis of Myc-tagged ZNF827 and ZNF827 ZnF mutant proteins in U-2 OS ZNF827 knockout cells 48 h after transfection. **(F)** Western blot analysis of RPA32 and NuRD components HDAC1 and MTA1, following co-IP with a direct ZNF827 antibody in HT1080 cells overexpressing ZNF827. Source data are provided in the accompanying Source Data file.

Supplementary Figure 2.

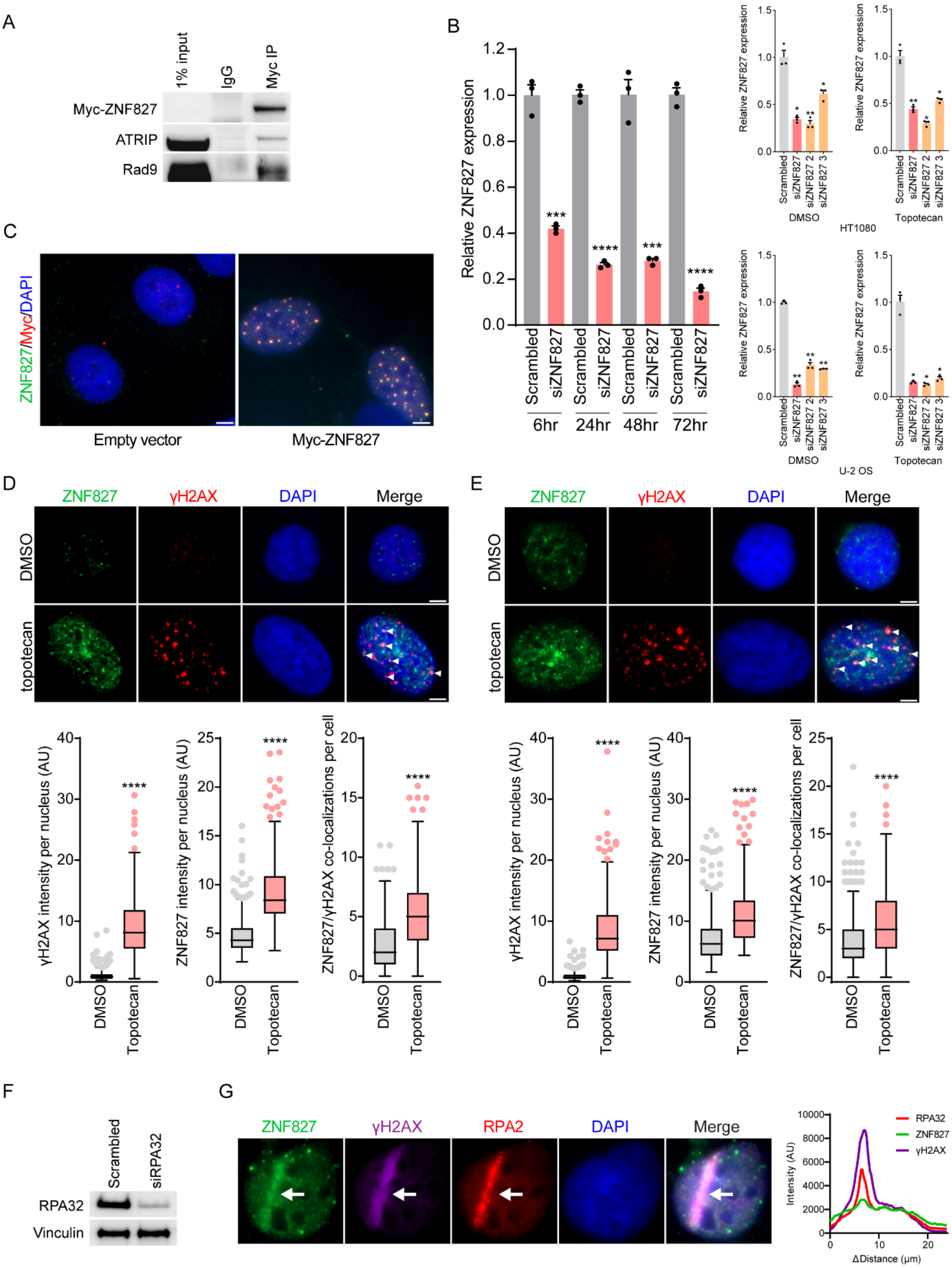

Supplementary Figure 2. ZNF827 localizes to DNA damage sites. Related to Figure 2. (A) Western blot of ATRIP, Rad9 and Myc following treatment with benzonase and co-immunoprecipitation (co-IP)

in U-2 OS cells overexpressing Myc-tagged ZNF827. **(B)** qRT-PCR validation of ZNF827 knockdown at 6, 24, 48 and 72 h post siRNA transfection in U-2 OS cells (left panel). qRT-PCR validation of ZNF827 knockdown 72 h post siRNA transfection using three different ZNF827 siRNAs in U-2 OS and HT1080 cells (right panel). Data normalised to scrambled controls and presented as mean + SEM; * $P<0.01$; ** $P<0.005$; *** $P<0.0002$; **** $P<0.0001$ by two tailed *t* test. **(C)** Representative images showing ZNF827 antibody verification by immunofluorescence in U-2 OS cells overexpressing Myc-tagged ZNF827. **(D)** Representative images of γH2AX (red) and endogenous ZNF827 (green) foci in HT1080 cells (top panel). White arrows indicate colocalizations. Scale bar represents 5 μm. Quantitation of γH2AX integrated intensity per nucleus, ZNF827 integrated intensity per nucleus, and γH2AX and ZNF827 colocalizations per nucleus (bottom panel). Out of two independent experiments, n=494 DMSO and n=334 topotecan treated cells were assayed using CellProfiler. At least 50% overlap criterion was applied for colocalization count. Data are presented as Tukey box plots; **** $P<0.0001$ by Mann-Whitney tests. **(E)** Representative images of γH2AX (red) and endogenous ZNF827 (green) foci in HT1080 6TG cells (top panel). White arrows indicate colocalizations. Scale bar represents 5 μm. Quantitation of γH2AX integrated intensity per nucleus, ZNF827 integrated intensity per nucleus, and γH2AX and ZNF827 colocalizations per nucleus (bottom panel). Out of two independent experiments, n=554 DMSO and n=261 topotecan treated cells were assayed using CellProfiler. At least 50% overlap criterion was applied for colocalization count. Data are presented as Tukey box plots; **** $P<0.0001$ by Mann-Whitney tests. **(F)** Western blot analysis of RPA32 in U-2 OS cells 72 h post RPA32 knockdown. **(G)** Representative images showing colocalizations of laser stripes of ZNF827 (green), γH2AX (purple) and RPA32 (red) in HT1080 cells (left panel). Corresponding intensity profile of ZNF827 (green), γH2AX (purple) and RPA32 (red) (right panel). Profile generated using ZEN (Zeiss) software. Source data are provided in the Source Data file.

Supplementary Figure 3.

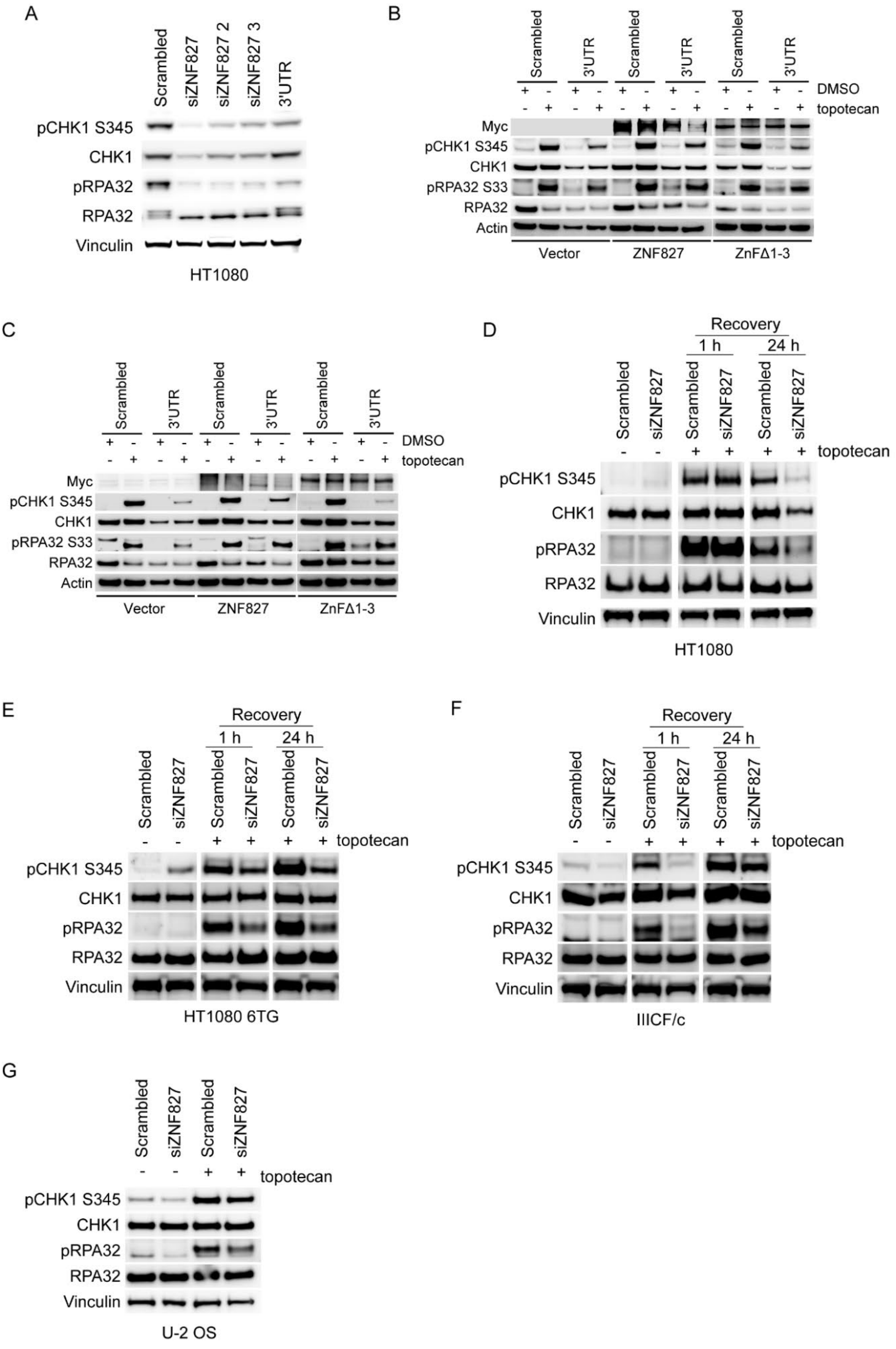

Supplementary Figure 3. ZNF827 depletion abrogates ATR signalling. Related to Figure 3. (A) Western blot analysis of pCHK1 (S345), total CHK1, pRPA32 (S33) and total RPA32 in HT1080 cells 72 h post ZNF827 knockdown with three different siRNAs, an siRNA targeting the 3′ UTR of ZNF827, and a scrambled siRNA control. Vinculin used as a loading control. **(B, C)** Replicate western blot analysis of Myc, pCHK1 (S345), total CHK1, pRPA32 (S33) and total RPA32 in U-2 OS cells stably expressing Myc-tagged ZNF827, Myc-tagged ZNF827 ZnFΔ1-3 or vector control. Endogenous ZNF827 was depleted using a ZNF827 3' UTR siRNA and a scrambled siRNA was used as a control. 72 h after the addition of siRNA, cells were treated with 2 µg/mL topotecan or DMSO followed by 1 h recovery. Actin used as a loading control. **(D)** Western blot analysis of pCHK1 (S345), total CHK1, pRPA32 (S33) and total RPA32 in HT1080 cells 72 h post ZNF827 knockdown, including 1 h treatment with 2 µg/mL topotecan or DMSO followed by 1 h or 24 h recovery. Vinculin used as a loading control. **(E)** Western blot analysis of pCHK1 (S345), total CHK1, pRPA32 (S33) and total RPA32 in HT1080 6TG cells 72 h post ZNF827 knockdown, including 1 h treatment with 2 µg/mL topotecan or DMSO followed by 1 h or 24 h recovery. Vinculin used as a loading control. **(F)** Western blot analysis of pCHK1 (S345), total CHK1, pRPA32 (S33) and total RPA32 in IICF/c cells 72 h post ZNF827 knockdown, including 1 h treatment with 2 µg/mL topotecan or DMSO followed by 1 h or 24 h recovery. Vinculin used as a loading control. **(G)** Western blot analysis of pCHK1 (S345), total CHK1, pRPA32 (S33) and total RPA32 in U-2 OS cells, 24 h post transfection with ZNF827 siRNA, including 1 h incubation with 2 µg/mL topotecan or DMSO followed by 1 h recovery. Source data are provided in the accompanying Source Data file.

Supplementary Table 1: Mass spectrometry analysis of ZNF827 pulldown by HaloTag®

Accession	Description	Sequence coverage	Score	Num. of sequences
ZN827_HUMAN	Zinc finger protein 827 OS=Homo sapiens GN=ZNF827 PE=1 SV=1	0.46	4478	36
AP2A2_HUMAN	AP-2 complex subunit alpha-2 OS=Homo sapiens GN=AP2A2 PE=1 SV=2	0.05	110	2
CH60_HUMAN	60 kDa heat shock protein, mitochondrial OS=Homo sapiens GN=HSPD1 PE=1 SV=2	0.09	90	3
RLA2_HUMAN	60S acidic ribosomal protein P2 OS=Homo sapiens GN=RPLP2 PE=1 SV=1	0.17	83	2
MIF_HUMAN	Macrophage migration inhibitory factor OS=Homo sapiens GN=MIF PE=1 SV=4	0.08	74	1
TBA1A_HUMAN	Tubulin alpha-1A chain OS=Homo sapiens GN=TUBA1A PE=1 SV=1	0.03	63	1
RS27A_HUMAN	Ubiquitin-40S ribosomal protein S27a OS=Homo sapiens GN=RPS27A PE=1 SV=2	0.1	63	1
VWA8_HUMAN	von Willebrand factor A domain-containing protein 8 OS=Homo sapiens GN=VWA8 PE=1 SV=2	0.01	35	1
H2B1B_HUMAN	Histone H2B type 1-B OS=Homo sapiens GN=HIST1H2BB PE=1 SV=2	0.12	35	1
MELT_HUMAN	Ventricular zone-expressed PH domain-containing protein homolog 1 OS=Homo sapiens GN=VEPH1 PE=2 SV=1	0.01	34	1
STX11_HUMAN	Syntaxin-11 OS=Homo sapiens GN=STX11 PE=1 SV=1	0.02	31	1
CLCF1_HUMAN	Cardiotrophin-like cytokine factor 1 OS=Homo sapiens GN=CLCF1 PE=1 SV=1	0.04	28	1
L2GL1_HUMAN	Lethal(2) giant larvae protein homolog 1 OS=Homo sapiens GN=LLGL1 PE=1 SV=3	0.01	26	1
EGF_HUMAN	Pro-epidermal growth factor OS=Homo sapiens GN=EGF PE=1 SV=2	0.03	23	1
SMCA2_HUMAN	Probable global transcription activator SNF2L2 OS=Homo sapiens GN=SMARCA2 PE=1 SV=2	0.02	22	1
TX13C_HUMAN	Testis-expressed sequence 13C protein OS=Homo sapiens GN=TEX13C PE=5 SV=1	0.04	22	1
NAA15_HUMAN	N-alpha-acetyltransferase 15, NatA auxiliary subunit OS=Homo sapiens GN=NAA15 PE=1 SV=1	0.04	21	1
ITA2B_HUMAN	Integrin alpha-IIb OS=Homo sapiens GN=ITGA2B PE=1 SV=3	0.02	21	1
CCD57_HUMAN	Coiled-coil domain-containing protein 57 OS=Homo sapiens GN=CCDC57 PE=1 SV=2	0.01	19	1
KLRB1_HUMAN	Killer cell lectin-like receptor subfamily B member 1 OS=Homo sapiens GN=KLRB1 PE=1 SV=1	0.04	19	1
GEMI_HUMAN	Geminin OS=Homo sapiens GN=GMNN PE=1 SV=1	0.04	18	1
VIME_HUMAN	Vimentin OS=Homo sapiens GN=VIM PE=1 SV=4	0.04	17	1
COLA1_HUMAN	Collagen alpha-1(XXI) chain OS=Homo sapiens GN=COL21A1 PE=2 SV=1	0.02	16	1
PTH2R_HUMAN	Parathyroid hormone 2 receptor OS=Homo sapiens GN=PTH2R PE=1 SV=1	0.01	16	1
IGS10_HUMAN	Immunoglobulin superfamily member 10 OS=Homo sapiens GN=IGSF10 PE=2 SV=1	0	16	1
RM15_HUMAN	39S ribosomal protein L15, mitochondrial OS=Homo sapiens GN=MRPL15 PE=1 SV=1	0.05	16	1
CCNI_HUMAN	Cyclin-I OS=Homo sapiens GN=CCNI PE=2 SV=1	0.04	16	1
EVL_HUMAN	Ena/VASP-like protein OS=Homo sapiens GN=EVL PE=1 SV=2	0.03	15	1
BUB1B_HUMAN	Mitotic checkpoint serine/threonine-protein kinase BUB1 beta OS=Homo sapiens GN=BUB1B PE=1 SV=3	0.01	15	1
SPEF2_HUMAN	Sperm flagellar protein 2 OS=Homo sapiens GN=SPEF2 PE=2 SV=2	0	15	1
SOX18_HUMAN	Transcription factor SOX-18 OS=Homo sapiens GN=SOX18 PE=1 SV=2	0.04	14	1
FBX16_HUMAN	F-box only protein 16 OS=Homo sapiens GN=FBXO16 PE=2 SV=1	0.06	14	1

Supplementary Table 2 – List of antibodies

Target	Species	Manufacturer	Catalogue No.	Dilution
**Actin**	Rabbit	Sigma	A2066	**WB:** 1:5000 in PBST
**ATR**	Rabbit	CST	2790S	**WB:** 1:500 in PBST
**ATRIP**	Rabbit	CST	2737	**WB:** 1:1000 in PBST
**BrdU**	Rat	Abcam	Ab6326	**IF:** 1:500 in ABDIL
**BRCA1**	Mouse	GenTex	GTX70111	**IF:** 1:500 in ABDIL
**CHK1**	Mouse	CST	2360	**WB:** 1:1000 in PBST
**ETAA1**	Rabbit	Abcam	ab197017	**WB:** 1:1000 in PBST
**HDAC1**	Rabbit	CST	2062S	**WB:** 1:1000 in PBST
**IgG2a Isotype control**	Mouse	CST	61656	**IP:** 2.5ug per sample
**MTA1**	Rabbit	Abcam	ab71153	**WB:** 1:1000 in PBST
**Myc-tag**	Mouse	CST	2276S	**WB:** 1:1000 in PBST<br>**IF:** 1:500 in ABDIL<br>**IP:** 2.5ug per sample
**p21**	Rabbit	CST	2947S	**WB:** 1:1000 in PBST
**p53**	Mouse	CST	2524S	**WB:** 1:1000 in PBST
**pCHK1 S345**	Rabbit	CST	2348S	**WB:** 1:1000 in TBST
**pRPA32 S33**	Rabbit	Bethyl Laboratories	A300-246A	**WB:** 1:1000 in TBST<br>**IF:** 1:500 in ABDIL
**Rad9**	Rabbit	Abcam	ab70810	**WB:** 1:1000 in PBST
**RBBP4**	Rabbit	Abcam	ab1765	**WB:** 1:1000 in PBST
**RPA32**	Mouse	Abcam	ab2175	**WB:** 1:1000 in PBST
**RPA70**	Rabbit	Abcam	ab79398	**WB:** 1:1000 in PBST
**TOPBP1**	Rabbit	Abcam	Ab2402	**WB:** 1:1000 in PBST<br>**IF:** 1:500 in ABDIL
**Vinculin**	Mouse	Sigma-Merck	V9131	**WB:** 1:10 000 in PBST
**ZNF827**	Goat	Santa Cruz	T-20	**WB:** 1:500 in PBST<br>**IF:** 1:2500 in ABDIL
**γH2AX S139**	Rabbit	Abcam	Ab2893	**WB:** 1:1000 in PBST<br>**IF:** 1:500 in ABDIL
