# Supplementary material for: ZNF827 is a single-stranded DNA binding protein that regulates the ATR-CHK1 DNA damage response pathway
Source: Nat Commun. 2024 Mar 12;15:2210. doi: 10.1038/s41467-024-46578-0 (PMC10933417; doi:10.1038/s41467-024-46578-0)
Supplement: Supplementary file 3 — Description of Additional Supplementary Files [file 41467_2024_46578_MOESM3_ESM.pdf]

Description of Additional Supplementary Files

File Name: Supplementary Movie 1
Description: **ZNF827 colocalizes with RPA.** Live cell imaging of HaloTag-ZNF827 (red) and GFP-RPA32 (green) in U-2 OS cells.

File Name: Supplementary Movie 2
Description: **ZNF827 tracks with the replication machinery.** Live cell imaging of HaloTag-ZNF827 (red) and PCNA chromobodies (green) in U-2 OS cells.
